# Supplementary material for: Do customers’ perceptions of Islamic banking services predict satisfaction and word of mouth? Evidence from Islamic banks in Bangladesh
Source: PLoS One. 2023 Jan 20;18(1):e0280108. doi: 10.1371/journal.pone.0280108 (PMC9858845; doi:10.1371/journal.pone.0280108)
Supplement: Attachment — Submitted filename: Review of paper.docx [file pone.0280108.s002.docx]

Review of paper

Title of paper: **Do customers’ perceptions of Islamic banking services reflect word of mouth:**

**Evidence from Islamic banks in Bangladesh.**

I reviewed the above paper and submit the following suggestions for improvement of its quality: -

**1**. One of the main variables in this study is “words of mouth” which the authors have analyzed and tried to prove effective for future growth of Islamic banking services. But in the model it is shown as dependent variable. The authors must know that only independent variables have direct while mediating or moderating variables have indirect effect on dependent variable, which is always one. If the author wants to measure the impact of “Words of Mouth” on the satisfaction of customers and banking services, they should use it as an independent variable and measures its impact on expansion of Islamic banking.

**2**. According to authors, “security has a highly significant impact on customer perception of Islamic banking services, because Islamic banks endure updated operating systems with high-security patches and the matter of this high-security influences customers in using Islamic banking services.”

The author should clearly illustrate what type of distinct “operating system” Islamic banks has and how it is different from conventional banks as well as how it ensures the deposit, transactions and high return to customers.

**3**. The author stated that “the fifth author of this study physically distributed 700 questionnaires to the customers of Islamic banks in Bangladesh between the time period of April and July 2021. We
received 385 responses from the bank customers out of which we identified a total of 377 valid responses for data, 53.86% response rate of data collection.”

The author could not justify why the response rate was so much low when 700 questionnaires were physically distributed among respondents. The author must disclose why 315 questionnaires were not included into the analysis. Mostly the response rate is more than 80 percent when data is collected through survey method as the author has done. Here response rate is very low. It must be justified through valid arguments.

**4.** Capital R-square (R^2 )^ is used to show goodness of fit of the model and ratio of variation in the dependent variable on account of independent variables. But author wrote r-square to denote the model, which is not correct. The must understand difference between r-square and R-square.

**5.** The author could not explain the meaning of another important factor “Benefit” properly. They must have explained what is its meaning in Islamic banking and what types of benefits Islamic banks offer to their customers. Moreover, the empirical results also show negative association between benefit and perception of Islamic banking services. When the customers have negative perception about the benefit to be accrued from Islamic banks how can they refer or convince others to opt Islamic banking services through the “word of mouth”.

The authors have also failed to justify why all other studies such as Thambiah et al. (2011), Oladapo et al. (2021) and Saqib et al. (2016) conducted in Pakistan, Malaysia and Saudi Arabia have positive association between benefit and perception of Islamic banking services while their study has negative association between two variables.

**6.** The authors states that “the customers choose Islamic banking products and services as Islamic banks served the welfare of society and fulfilled their promises”.

They must have defined welfare role or services and promises of Islamic Banks because all banks whether Islamic or conventional are commercial business organization and their prime objective is to maximize their profit. Conventional Banks also fulfill their promises by giving fixed return to their depositors while Islamic banks do not make any promise about fixed return because they collect all deposits under profit and loss sharing mechanism. The author must illustrate this point for satisfaction of the customers of Islamic Banks and general readers of this paper.

7. There are grammatical errors which may be removed through proper editing by an English expert.

**Recommendations:** The paper needs minor revisions.

Best Regards

Prof. Dr. Abdul Ghafoor Awan

Dean,

Faculty of Management and Social Sciences,

Institute of Southern Punjab,Multan

Pakistan.

July 06,2022.
