# Supplementary material for: Do customers’ perceptions of Islamic banking services predict satisfaction and word of mouth? Evidence from Islamic banks in Bangladesh
Source: PLoS One. 2023 Jan 20;18(1):e0280108. doi: 10.1371/journal.pone.0280108 (PMC9858845; doi:10.1371/journal.pone.0280108)
Supplement: Attachment — Submitted filename: Review Report.docx [file pone.0280108.s004.docx]

Review Report

I again reviewed the paper entitled **“Do Customers’ perception of Islamic Banking services reflect word of mouth? Evidence from Islamic Banks in Bangladesh”,** suggest the following minor revisions: -

1. The author distributed 700 questionnaires among the respondents and received back 385 and out of them found 377 valid for analysis. The response rate was 53.86% and while the participants include 76.1% male and 23.9% female. He did not give mentioned the causes of low response and low participation from female side.

**2**.The author did not explain the mechanism of benefit to be received by customers of Islamic Banks in financial term theoretically or empirically as the customers of conventional banks get benefit in the form of fixed return.

3. No practical mechanism of security of investment in Islamic banks has been mentioned in the paper as the whole function of Islamic banks is based on profit and loss.

4.The heading of Table on page 12 is wrong. It is descriptive statistics of variables not Reliability test. Mostly reliability is checked through Cronbach Alpha, which is not applied in this paper.

**5.** At page 12 r-square, actually, is R^2^

6.Table 6 show path coefficient in which relationship between benefits and customer satisfaction is negative as their value is -0.088 and 0.071. The author did not give valid justification for this negative relationship or why customer have negative perception about benefits from Islamic banks although this relationship is positive in the studies conducted in Pakistan, Malaysia and Saudi Arabia.

Regards

Prof.Dr. Abdul Ghafoor Awan,

Dean,

Faculty of Management Science.

Institute of Southern Punjab, Multan
